# Supplementary material for: Youth Tobacco Control in the Digital Age: Impact of South Carolina’s Youth Tobacco Education and Vaping Cessation Social Media Programs
Source: Int J Environ Res Public Health. 2025 Feb 12;22(2):269. doi: 10.3390/ijerph22020269 (PMC11855303; doi:10.3390/ijerph22020269)
Supplement: Supplementary file 1 [file ijerph-22-00269-s001.zip › ijerph-3384040-supplementary.pdf]

Down & Dirty* and *Behind The Haze

Questions asked in both surveys unless otherwise noted.

<u>*Screening / Demographics*</u>

What is your birthdate?
- [free response accepting date]

What is your zip code?
- [free response accepting 5 digits]

Are you Hispanic or Latino? Select yes if you are Mexican, Mexican American, Chicano, Puerto Rican, Cuban, Cuban American, or other Hispanic or Latino not listed here.
- Yes
- No

What is your race/ethnicity? Select all that apply.
- American Indian/Alaska Native
- Asian
- Black or African American
- Native Hawaiian or Other Pacific Islander
- White
- Other, please specify: ____

What is your current gender identity?
- Female
- Male
- Genderqueer / Gender non-conforming
- Trans female / Trans woman
- Trans male / Trans man
- Other, please specify: ______
- Prefer not to say

[*D&D* only] Rank the 3 people that would BEST FIT in your main group of friends, starting with the best fit: [female photos]
1. [photo # 1-40]
2. [photo # 1-40]
3. [photo # 1-40]

[*D&D* only] Rank the 3 people that would LEAST FIT in your main group of friends, starting with the worst fit: [female photos]
1. [photo # 1-40]
2. [photo # 1-40]
3. [photo # 1-40]

[*D&D* only] Rank the 3 people that would BEST FIT in your main group of friends, starting with the best fit: [male photos]

1. [photo # 41-80]
2. [photo # 41-80]
3. [photo # 41-80]

[*D&D* only] Rank the 3 people that would LEAST FIT in your main group of friends, starting with the worst fit: [male photos]

1. [photo # 41-80]
2. [photo # 41-80]
3. [photo # 41-80]

Tobacco Use

For the following questions, please indicate if you have ever tried…
Using chewing tobacco, snuff, or dip such as Redman, Levi Garrett, Beechnut, Skoal, Skoal Bandits, or Copenhagen, even just a little bit.

- Yes
- No

Smoking cigarettes, even one or two puffs.

- Yes
- No

Using vapes or e-cigarettes such as JUUL, SMOK, VUSE, Suorin, blu, Happy Sticks or Puff Bar, WITHOUT MARIJUANA, even one or two puffs.

- Yes
- No

Smoking traditional cigars, cigarillos, or little or filtered cigars such as Arturo Fuerte, Swisher Sweets, or Black and Mild, WITHOUT MARIJUANA, even one or two puffs.

- Yes
- No

Using vapes that contain marijuana, marijuana concentrations, marijuana waxes, THC, CBD, or hash oils.

- Yes
- No

In the past 30 days, on how many days did you use chewing tobacco, snuff, or dip such as Redman, Levi Garrett, Beechnut, Skoal, Skoal Bandits, or Copenhagen, even just a little bit?

- [free response accepting 0-30]

In the past 30 days, on how many days did you smoke at least one cigarette?

- [free response accepting 0-30]

In the past 30 days, on how many days did you use vapes or e-cigarettes such as JUUL, SMOK, VUSE, Suorin, blu, Happy Sticks or Puff Bar, WITHOUT MARIJUANA, even one or two puffs?

- [free response accepting 0-30]

In the past 30 days, on how many days did you use traditional cigars, cigarillos, or little or filtered cigars such as Arturo Fuerte, Swisher Sweets, or Black and Mild, WITHOUT MARIJUANA, even one or two puffs?

- [free response accepting 0-30]

In the past 30 days, on how many days did you use vapes that contain marijuana, marijuana concentrations, marijuana waxes, THC, CBD, or hash oils?

- [free response accepting 0-30]

Tobacco Use Susceptibility

[Asked to participants who reported no use of the product]

Have you ever been curious about using a vape or electronic cigarette (e-cigarette)?

- Definitely yes
- Probably yes
- Probably not
- Definitely not

If one of your best friends were to offer you a vape or an electronic cigarette (e-cigarette), would you use it?

- Definitely yes
- Probably yes
- Probably not
- Definitely not

Do you think that you will use a vape or an electronic cigarette (e-cigarette) soon?

- Definitely yes
- Probably yes
- Probably not
- Definitely not

Do you think you will use a vape or an electronic cigarette (e-cigarette) at any time in the next year?

- Definitely yes
- Probably yes

- Probably not
- Definitely not

[*D&D* only] Have you ever been curious about chewing tobacco?
- Definitely yes
- Probably yes
- Probably not
- Definitely not

[*D&D* only] If one of your best friends were to offer you chewing tobacco, would you chew it?
- Definitely yes
- Probably yes
- Probably not
- Definitely not

[*D&D* only] Do you think that you will use chewing tobacco soon?
- Definitely yes
- Probably yes
- Probably not
- Definitely not

[*D&D* only] Do you think you will use chewing tobacco at any time in the next year?
- Definitely yes
- Probably yes
- Probably not
- Definitely not

[*D&D* only] Have you ever been curious about smoking a cigarette?
- Definitely yes
- Probably yes
- Probably not
- Definitely not

[*D&D* only] If one of your best friends were to offer you a cigarette, would you smoke it?
- Definitely yes
- Probably yes
- Probably not
- Definitely not

[*D&D* only] Do you think that you will smoke a cigarette soon?
- Definitely yes
- Probably yes
- Probably not
- Definitely not

[*D&D* only] Do you think you will smoke a cigarette at any time in the next year?
- Definitely yes
- Probably yes
- Probably not
- Definitely not

Knowledge - Down and Dirty Only

Have you heard of this statement before? Dipping and chewing tobacco leads to tooth loss and gum disease.
- Yes
- No

Have you heard of this statement before? If you use tobacco, your younger siblings or relatives are more likely to also use tobacco.
- Yes
- No

Have you heard of this statement before? 1,300 Americans die each day from tobacco use.
- Yes
- No

Have you heard of this statement before? Vape aerosol contains up to 31 chemicals like nickel, lead, and benzene that can damage your lungs.
- Yes
- No

Have you heard of this statement before? Dip and chewing tobacco can cause mouth cancer.
- Yes
- No

Have you heard of this statement before? If you quit chewing tobacco, you can save about $1,000 a year.
- Yes
- No

Have you heard of this statement before? Nicotine changes your brain in a way that affects your mood, making you not feel right.
- Yes
- No

Have you heard of this statement before? Chemicals in chew and dip are absorbed through your mouth, which can cause cancer.

- Yes
- No

Have you heard of this statement before? Teens who use vapes can spend over $700 in six months on one pack of pods or three disposables per week, which is about $1,500 a year.
- Yes
- No

Have you heard of this statement before? Vape aerosol contains toxic chemicals like arsenic, lead, and formaldehyde, which even in small amounts, can cause brain damage, lung disease, or cancer.
- Yes
- No

Knowledge - Behind the Haze Only

Have you heard of this statement before? The chemicals in vapes break down the defenses in your lungs, making you more vulnerable to viruses.
- Yes
- No

Have you heard of this statement before? Even if you are strong, young, and healthy, the chemicals in vapes can weaken you and damage your lungs on a cellular level.
- Yes
- No

Have you heard of this statement before? Vape aerosols contain lead, a neurotoxin that can cause brain damage.
- Yes
- No

Have you heard of this statement before? Vape juice and vapor contain dangerous chemicals, like formaldehyde. Formaldehyde is used to preserve dead bodies.
- Yes
- No

Have you heard of this statement before? Vaping can cause a chemical burn in the lungs, similar to that seen in people exposed to poisons like mustard gas.
- Yes
- No

Have you heard of this statement before? Vape companies went into schools and lied to teens, telling them that vapes are “totally safe.”
- Yes

- No

Have you heard of this statement before? Nicotine can mess with neurotransmitters in the brain that are linked to stress, anxiety & depression.
- Yes
- No

Have you heard of this statement before? Nicotine affects the natural balance of neurotransmitters in the brain such as dopamine and serotonin, which are important for stabilizing mood.
- Yes
- No

Have you heard of this statement before? Sharing vapes with friends also means sharing nicotine addiction.
- Yes
- No

Attitudes & Beliefs - Down and Dirty Only

How much do you agree with the following statements?
Chewing tobacco is dangerous to a person's health.
- Strongly Disagree
- Disagree
- Neither Agree nor Disagree
- Agree
- Strongly Agree

Chewing tobacco is a waste of money.
- Strongly Disagree
- Disagree
- Neither Agree nor Disagree
- Agree
- Strongly Agree

It is important to me to live a tobacco-free lifestyle.
- Strongly Disagree
- Disagree
- Neither Agree nor Disagree
- Agree
- Strongly Agree

Using vapes and e-cigarettes is dangerous to a person’s health.
- Strongly Disagree

- Disagree
- Neither Agree nor Disagree
- Agree
- Strongly Agree

<u>Attitudes & Beliefs - Behind The Haze Only</u>

How much do you agree with the following statements?
If I were to use a vape, I would worry about my health risks.
- Strongly Disagree
- Disagree
- Neither Agree nor Disagree
- Agree
- Strongly Agree

If I were to use a vape, I would harm my lungs.
- Strongly Disagree
- Disagree
- Neither Agree nor Disagree
- Agree
- Strongly Agree

If I were to use a vape, I would worry about the chemicals that I am inhaling.
- Strongly Disagree
- Disagree
- Neither Agree nor Disagree
- Agree
- Strongly Agree

If I were to use a vape, I would put myself at risk for addiction.
- Strongly Disagree
- Disagree
- Neither Agree nor Disagree
- Agree
- Strongly Agree

If I were to use a vape, I would worry about how it would affect my brain.
- Strongly Disagree
- Disagree
- Neither Agree nor Disagree
- Agree
- Strongly Agree

If I were to use a vape, I would worry about how it would affect my emotions.

- Strongly Disagree
- Disagree
- Neither Agree nor Disagree
- Agree
- Strongly Agree

Campaign Awareness & Reception

Have you heard or seen of the following brands?
Behind the Haze
- Yes
- No
- I don't know

Down and Dirty
- Yes
- No
- I don't know

How much do you like [Down and Dirty/Behind the Haze]?
- I really don't like it
- I don't like it
- Neither like nor dislike it
- I like it
- I really like it

Video Advertisements

[Participant views video advertisement]

Have you seen this video before?
- Yes
- No
- I'm not sure

On a scale of 1 to 5, how would you rate the claims or arguments in this advertisement:
- 1 NOT BELIEVABLE
- 2 Slightly believable
- 3 Moderately believable
- 4 Very believable
- 5 Extremely BELIEVABLE

How much do you agree with the following statements?
This advertisement grabbed my attention.

- Strongly Disagree
- Disagree
- Neither Agree nor Disagree
- Agree
- Strongly Agree

This advertisement is convincing.
- Strongly Disagree
- Disagree
- Neither Agree nor Disagree
- Agree
- Strongly Agree

This advertisement is informative.
- Strongly Disagree
- Disagree
- Neither Agree nor Disagree
- Agree
- Strongly Agree

This advertisement is meaningful to me.
- Strongly Disagree
- Disagree
- Neither Agree nor Disagree
- Agree
- Strongly Agree

This advertisement is powerful.
- Strongly Disagree
- Disagree
- Neither Agree nor Disagree
- Agree
- Strongly Agree

This advertisement is worth remembering.
- Strongly Disagree
- Disagree
- Neither Agree nor Disagree
- Agree
- Strongly Agree

[Section repeated for each video advertisement]

Quit The Hit

Baseline Only

What is your age?

- [free response accepting numbers]

What is your zip code?

- [free response accepting 5 digits]

Are you Hispanic or Latino? Select yes if you are Mexican, Mexican American, Chicano, Puerto Rican, Cuban, Cuban American, or other Hispanic or Latino not listed here.

- Yes
- No

What is your race? (Check all that apply)

- American Indian or Alaska Native
- Asian
- Black or African American
- Native Hawaiian or Other Pacific Islander
- White
- Other, please specify: ______
- Prefer not to answer

What is your current gender identity? (Select all that apply).

- Female
- Male
- Genderqueer / gender non-conforming
- Trans woman
- Trans man
- Different identity, please specify: _______
- Prefer not to say

Which of the following are reasons that you joined the Quit the Hit program? (Select all that apply).

- I can access the group online
- It provides expert support
- I can talk to my peers/ people who also want to quit
- I can get money for taking the surveys
- Another reason (please specify): ______

Baseline, Follow-up 1, and Follow-up 2

In the past 30 days, on how many days did you use a nicotine vape?

- [free response accepting 0-30]

In the past 30 days, on how many days did you smoke at least one cigarette?
- [free response accepting 0-30]

In the past 30 days, on how many days did you use little cigars or cigarillos (e.g. Black and Mild, Swisher Sweets) but WITHOUT MARIJUANA and NOT FOR BLUNTS?
- [free response accepting 0-30]

In the past 30 days, on how many days did you use spit tobacco, chew, dip, or moist snuff (e.g. Copenhagen, Skoal, Grizzly)?
- [free response accepting 0-30]

In the past 30 days, on how many days did you use a marijuana or cannabis vape?
- [free response accepting 0-30]

Now, we'd like to know how successful you expect to be quitting vaping nicotine at this time. Do you think you could do it? BE REALISTIC ABOUT THIS, based on your past experiences and your present strength of motivation.
- 1 - Lowest expectation of success
- 2
- 3
- 4
- 5
- 6
- 7
- 8
- 9
- 10 - Highest expectation of success

How confident are you that you can stay completely NICOTINE free in the next 6 months?
- Not at all confident
- Slightly confident
- Moderately confident
- Very confident
- Extremely confident

Follow-up 1 and/or 2 Only
Please rate each statement using the following scale. Select one for each statement.

The messages from the facilitator were easy to understand.
- Strongly Disagree
- Disagree
- Neither Agree nor Disagree
- Agree
- Strongly Agree

I believe the facilitator gave sound advice.
- Strongly Disagree
- Disagree
- Neither Agree nor Disagree
- Agree
- Strongly Agree

I would recommend this program to others.
- Strongly Disagree
- Disagree
- Neither Agree nor Disagree
- Agree
- Strongly Agree

The messages from the facilitator gave me something new to think about.
- Strongly Disagree
- Disagree
- Neither Agree nor Disagree
- Agree
- Strongly Agree

The messages from the facilitator have helped me to be healthier.
- Strongly Disagree
- Disagree
- Neither Agree nor Disagree
- Agree
- Strongly Agree

I have used information shared in the group.
- Strongly Disagree
- Disagree
- Neither Agree nor Disagree
- Agree
- Strongly Agree

I have thought about what I read in the group.
- Strongly Disagree
- Disagree
- Neither Agree nor Disagree
- Agree
- Strongly Agree

I tried to take action on the suggestions / quit tips shared in the group.

- Strongly Disagree
- Disagree
- Neither Agree nor Disagree
- Agree
- Strongly Agree

Please indicate how many of the 30 daily Instagram messages from the facilitator to the Instagram group you read.

- None (0)
- Only a few (1-5)
- Some of them (6-12)
- About half of them (13-19)
- Most of them (20-29)
- All of them (30)

Will you refer back to the content posted in the Instagram group for more information and ideas for changing your vaping?

- Definitely yes
- Probably yes
- Might or might not
- Probably not
- Definitely not

Since you completed the Quit the Hit program, did you refer back to the content posted in the Instagram group or on the profile page for more information and ideas for changing your vaping?

- Yes
- No
- Not sure / don’t remember
